# Exploring the understanding of how parenting influences the children's nutritional status, physical activity, and BMI

**DOI:** 10.3389/fnut.2022.1096182

**Published:** 2023-01-11

**Authors:** Betül Kocaadam-Bozkurt, Saniye Sözlü, Melahat Sedanur Macit-Çelebi

**Affiliations:** ^1^Department of Nutrition and Dietetics, Faculty of Health Sciences, Erzurum Technical University, Erzurum, Turkey; ^2^Department of Nutrition and Dietetics, Faculty of Health Sciences, Tokat Gaziosmanpaşa University, Tokat, Turkey; ^3^Department of Nutrition and Dietetics, Faculty of Health Sciences, Ondokuz Mayis University, Samsun, Turkey

**Keywords:** child, family, parenting, nutrition, obesity, physical activity

## Abstract

**Aim:**

Parental behaviors and the home environment are two of the most effective ways to adopt healthy eating and active lifestyles. For this reason, it is crucial to understand children's nutritional habits, analyze the dynamics related to parental factors, diagnose and treat childhood obesity in the early period, and prevent adulthood obesity. This study aimed to explore how parenting influences children's nutritional status, physical activity, and BMI.

**Methods:**

The study involved 596 children with their parents. The data were collected through face-to-face interviews using the survey method. The survey consists of descriptive information (age, gender, educational status), anthropometric measurements, nutritional habits, Family Nutrition and Physical Activity Scale (FNPA), International Physical Activity Questionnaire, and 24-h dietary recall. The Mean Adequacy Ratio (MAR) was applied to assess dietary adequacy.

**Results:**

Most mothers and fathers were overweight or obese (61.6 and 68.7%, respectively). 38.6% of boys and 23.1% of girls were overweight or obese. The FNPA score was positively correlated with MAR (*p* < 0.05). Multiple linear regression analysis revealed that children's BMI was negatively correlated with FNPA score, while maternal BMI and father's BMI were positively correlated (p < 0.05). Furthermore, dietary energy was not associated with the child's BMI but with dietary adequacy (p < 0.05). There was no evidence that family impacted children's physical activity.

**Conclusion:**

This study supports that parenting influences children's dietary intake and BMI. Adequate and balanced nutrition, regardless of dietary energy, may affect children's body weight. Family plays a significant role in influencing and forming children's lifestyle-related behaviors. Children's healthy eating and physical exercise habits can be encouraged through school-based programs involving families.

## 1. Introduction

Obesity is a chronic disease affected by both genetic and environmental factors (1). One of the most severe public health issues in the 21st century is childhood obesity, and the increase in its prevalence is dramatic (2). Obese children are more prone to becoming obese in adulthood (3). According to the Centers for Disease Control and Prevention (CDC), obesity prevalence is 12.7% among children aged 2–5, 20.7% among aged 6–11, and 22.2% among youth 12–19 years old in 2017–2020 (4). The World Health Organization (WHO) reported that in 2016, more than 340 million overweight or obese children and adolescents aged 5 to 19. The number of overweight and obese children worldwide is estimated to reach 70 million by 2025 (5). Although obesity is a multifactorial disease, it is known that an obesogenic environment contributes to the development of obesity by causing excessive eating behavior and physical inactivity. The family environment can affect the child's nutrition and physical activity habits; negative attitudes and behaviors can pave the way for the development of an obesogenic environment (6). For this reason, it is of utmost importance to children's nutritional habits to analyze the dynamics related to parental factors, diagnose and treat childhood obesity in the early period, and prevent adulthood obesity (7).

Parental behaviors and the home environment are the most effective ways to acquire healthy eating and active lifestyles associated with eating habits and obesity (7, 8). This effect might occur directly and indirectly, and the family's attitudes, behaviors, and beliefs play a role in this interaction (9). While adults make their own choices regarding their eating habits and physical activity, children do not have the opportunity to make this choice for themselves (10). Parents also shape the child's home food environment and thoughts about food (7, 11). Thus, families lay the foundations for children's eating behavior and habits (12). While sharing meals with children, encouraging healthy snacks, and teaching the benefits of fruit and vegetable consumption have made a positive impression, consuming more energy-dense foods with high fat and sugar content, such as fried foods, soft drinks, and sweets rather than food prepared at home would have the opposite effect on the child's eating behavior (13). Unhealthy eating habits based on this food environment cause various health problems, especially obesity (10).

Different tools have been developed which examine the impact of parental factors on the assessment of obesity (6, 14). The Family Nutrition and Physical Activity Screening Tool (FNPA) developed by Ihmels et al. is an easy-to-use screening tool that evaluates family environmental and behavioral factors by combining information from a range of behaviors such as family meals, TV in the bedroom, and the parental modeling of physical activity relates to childhood obesity (6). In studies, the Family Nutrition and Physical Activity (FNPA) screening tool has been shown to be significantly associated with obesity (6, 10, 14) and an increased odds of children at risk for being obese (15). Furthermore, as well as parenting behaviors, parents with high BMI scores (overweight or obese) had 2.18 times the odds (95% CI 1.11–4.27) of being in the low FNPA scores (less healthy environment) (16). In an up-to-date systematic review and meta-analysis, authors reported that a significant relationship was found between parental and child obesity (7). These study results indicate a multifactorial relationship between parental factors and the child's nutritional status.

Children's eating and physical activity habits can be influenced by their parents, who serve as significant social role models (16). Thus, evaluating the correlation between parenting and child nutrition is essential in revealing the factors affecting the development of healthy eating habits from an early age and preventing adult obesity. In this direction, this research aimed to evaluate the effect of the family on the child's nutrition, BMI, and physical activity level. To the best of our knowledge, this is the first study in Turkey in which the parental effect on children's nutritional status, physical activity, and BMI were comprehensively evaluated.

## 2. Material and methods

### 2.1. Study setting and participants

The study sample consisted of primary, secondary, and high school students aged 6–18 and their parents in Erzurum/Turkey. Participants were selected by simple random sampling from schools in Erzurum (one of the metropolitan cities of Turkey). The data were collected through face-to-face interviews using the survey method. Ethical permission was obtained from the Erzurum Technical University Ethics Committee (Meeting Number:10; Decision Number: 11; 20.04.2021) and the Erzurum Provincial Directorate of National Education (31.05.2021). The power analysis based on Al Yazeedi et al.'s study aimed to reach 385 children at a 5% type 1 error level and a confidence interval of 80% and 0.25 effect size (17). The study was completed with 596 children with their parents. The study was conducted according to the rules delineated in the Helsinki Declaration, and written informed consent from parents and children assent was obtained.

### 2.2. Measures

The survey consisted of descriptive information (age, gender, educational status), anthropometric measurements, nutritional habits, Family Nutrition and Physical Activity Scale (FNPA) (6, 9), International Physical Activity Questionnaire (18, 19), and 24-hour dietary recall. Energy and nutrient intakes of children were evaluated using the Nutrition Information System (BeBiS) program (The Food Code and Nutrient Data Base, BLS II.3, 1999, version 9.0).

### 2.3. Family nutrition and physical activity scale

The Family Nutrition and Physical Activity Scale was developed by Ihmel et al. with the Academy of Nutrition and Dietetics (American Dietetics Association) as a screening tool (for 6–18 years old) that evaluates family environments and combines information from various behaviors affiliated with childhood obesity. It can be used by nutrition researchers and professionals in clinical and public health (6). The reliability and validity study of the Turkish adaptation of the scale was conducted by Özdemir et al. (9). The scale consists of 20 items and five subscales (Physical activity, Parental behaviors, Unhealthy eating behaviors, Healthy nutrition, and Sedentary behaviors). It is evaluated in a four-point Likert type. Each item is scored as 1 (never/almost never), 2 (sometimes), 3 (often), and 4 (very often/always). The six items (3, 4, 5, 7, 10, and 13) are reverse coded. The total score obtained from the scale ranges from 20 to 80. Since there is no cut-off value, high scores show less risky family practices and child behaviors for the child's obesity. In contrast, low scores indicate a high-risk family environment, practices, and child behaviors. The scale's internal consistency (Cronbach's alpha) coefficient was determined to be 0.76 (9).

### 2.4. International physical activity questionnaire

The International Physical Activity Questionnaire (IPAQ) was developed by Craig et al. (18). The reliability and validity study of the Turkish adaptation of the IPAQ was conducted by Saglam et al. (19). It consists of seven questions and provides data on sitting, walking, engaging in moderate-intensity activity, and engaging in vigorous activity. All activities require that each activity be completed for at least 10 min at a time. By multiplying the minute, day, and MET (metabolic equivalent) value, a score is calculated as “MET-minutes/week”. Individuals are classified according to their physical activity level as low (< 600 MET-min/week), moderate (600-3000 MET-min / week), and high (> 3,000 MET-min / week) (18, 19).

### 2.5. Anthropometric measurements

The researcher measured the child's height and weight following the techniques described by Lohman et al. (20). The body weight and height of the parents were based on their self-reports. The WHO AnthroPlus software (version 1.0.4, February 2011) program were applied to evaluate the weight, height, Body Mass Index (BMI), and Z-scores of children. The BMI was categorized according to the Z-score junctions (21). For parents, Body Mass Index (BMI) value was calculated by dividing the body weight by the square of the height. BMI below 18.50 kg/m^2^ was classified as underweight, between 18.50–24.99 kg/m^2^ as normal, between 25.0–29.99 kg/m^2^ as overweight, and above 30.0 kg/m^2^ as obese (22).

### 2.6. Dietary adequacy

Twenty four hour dietary recall of the children was taken. The Mean Adequacy Ratio (MAR) was used to assess dietary adequacy using the Nutrient Adequacy Ratio (NAR). NAR was summed by analyzing individual daily consumption of nutrients with Dietary Reference Intake (DRI) rates categorized by gender and age. In the current study, a total of twelve nutrients, including vitamin B12, protein, vitamin B6, calcium, iron, fiber, folate, vitamin C, phosphorus, magnesium, potassium, and zinc, were selected as they are thought to be important in child nutrition, and NAR were calculated as a percentage (Formula 1) (23).


$$\begin{array}{l} NAR\left(\%\right)=\frac{Daily\;dietary\;intake\;of\;a\;nutrient}{\begin{array}{c} Dietary\;Reference\;Intake\\ recommendation\;of\;the\;nutrient \end{array}}\times 100 \end{array}$$

The Mean Adequacy Ratio was summed in percentage by taking the average of the NAR calculated for twelve nutrients (Formula 2). The diets of individuals are classified as inadequate ( ≤ 50 points), needing improvement (51–80 points), or good (>80 points) (23).


$$\begin{array}{l} MAR(\%)=\frac{\sum \;NAR(\%)}{Number\;of\;nutrients} \end{array}$$

### 2.7. Data analysis

The data were analyzed using SPSS 22.0. Normality test was performed to determine whether the parametric test assumptions were met. Descriptive statistical variables [mean and standard deviation] were used to analyze the data, divided into four tertiles according to the FNPA. The *t*-test, ANOVA test or Chi-squared test were applied to find value differences between groups. Factors that may be associated with a child's BMI (FNPA scores, dietary energy, MAR, MET, mother and father BMI) were evaluated using the multiple linear regression analysis. The relationship between the variables was evaluated using the Pearson correlation coefficient, and the outcome was assessed at a 95% confidence interval. The statistical significance level was set at p < 0.01 and p < 0.05.

## 3. Results

Descriptive information about the study sample is given in Tables 1, 2. The study comprised 596 children, 36.1% boys (13.3±2.4 years old) and 63.9% girls (13.6 ± 2.6 years old). The mean age of mothers was 41.5 ± 5.9 years, and of fathers was 46.4 ± 6.3 years. Most mothers and fathers were overweight or obese according to their BMI values (61.6 and 68.7%, respectively). When evaluated according to their educational status, most mothers (45.0%) were primary school graduates, while most fathers (46.9%) were high school graduates. 38.6% of boys and 23.1% of girls were overweight or obese. 55.2% of the children attended secondary school, and 39.6% attended high school. Physical activity was moderate in both boys and girls (600–3,000 MET-min / week). 28.4% of boys and 35.7% of girls had low physical activity levels. The mean FNPA score was 52.0 ± 8.3 (51.5 ± 7.3 in boys; 52.9 ± 8.3 in girls, *p* > 0.05). However, the Parental behaviors subscale score was higher in girls (Table 2).

**Table 1:** Descriptive characteristics of the parents, FNPA total and subscales scores (*n* = 596).

	**Father**	**Mother**	***p***
Age (years), mean ± SD	46.4 ± 6.3	41.5 ± 5.9	< 0.001^**^
BMI, mean ± SD	27.2 ± 3.8	27.1 ± 4.7	0.663
BMI class (n, %)			
Underweight	5 (0.8)	11 (1.8)	0.330
Normal weight	182 (30.5)	218 (36.6)	
Overweight	290 (48.7)	234 (39.3)	
Obese	119 (20.0)	133 (22.3)	
Education duration (years) mean ± SD (range)	11.4 ± 4.4	8.8 ± 4.7	< 0.001^**^
Education (n, %)			
Primary school graduate	119 (20.0)	268 (45.0)	< 0.001^**^
Secondary or high school graduate	279 (46.9)	217 (36.4)	
University/postgraduate	198 (33.1)	111 (18.6)	
FNPA total score	**X** **±SS**		
	52.0 ± 8.3		
Physical activity	11.2 ± 3.6		
Parental behaviors	9.8 ± 2.4		
Unhealthy eating behaviors	12.0 ± 2.3		
Healthy nutrition	8.3 ± 2.3		
Sedentary behaviors	10.5 ± 2.4		

BMI, body mass index; FNPA, family nutrition and physical activity scale.

** *p* < 0.001.

**Table 2:** Descriptive characteristics, BMI, physical activity levels, and FNPA scores according to gender.

	**Boys (*n* = 215)**	**Girls (*n* = 381)**	***p***
Age (years), mean ± SD	13.3 ± 2.4	13.6 ± 2.6	0.155
BMI, mean ± SD	20.4 ± 3.7	19.7 ± 3.6	0.020^*^
BMI class (n, %)			
Underweight	29 (13.5)	80 (21.0)	< 0.001^**^
Normal weight	103 (47.9)	213 (55.9)	
Overweight	58 (27.0)	69 (18.1)	
Obese	25 (11.6)	19 (5.0)	
Education (n, %)			
Primary school	13 (6.0)	18 (4.7)	0.413
Secondary school	124 (57.7)	205 (53.8)	
High school	78 (36.3)	158 (41.5)	
MET (minutes/week) mean ± SD	2,058.7 ± 826.1	1,567.7 ± 678.3	< 0.001^**^
MET classification (n, %)			
Inactive < 600	61 (28.4)	136 (35.7)	0.002^*^
Moderate active 600-3000	89 (41.4)	177 (46.5)	
Very active>3000	65 (30.2)	68 (17.8)	
FNPA total score	$\bar{\text{X}}$ **±SS**	$\bar{\text{X}}$ **±SS**	0.063
	51.5 ± 7.3	52.9 ± 8.3	
Physical activity	11.0 ± 3.5	11.6 ± 3.7	0.080
Parental behaviors	9.7 ± 2.5	10.2 ± 2.4	0.013^*^
Unhealthy eating behaviors	12.0 ± 2.0	12.1 ± 2.6	0.747
Healthy nutrition	8.3 ± 2.3	8.3 ± 2.4	0.833
Sedentary behaviors	10.6 ± 2.4	10.5 ± 2.4	0.909

BMI, Body Mass Index; FNPA, Family Nutrition and Physical Activity Scale; MET, Metabolic Equivalent.

* *p* < 0.05,

** *p* < 0.001.

Table 3 shows participants' age, BMI, and physical activity status according to quartile categories of the FNPA. In boys, the difference between quartile values only for age was significant (Q2–Q4) (*p* < 0.05). In girls, BMI value and age differed between Q1 and Q4 (*p* < 0.05). At the same time, maternal age, maternal BMI, and paternal age differed in the Q1–Q4 groups in girls (p < 0.05). MET values did not differ according to quartiles (*p* > 0.05).

**Table 3:** Evaluation of age, BMI, and physical activity status of participants according to quartile categories of the FNPA.

	**Boys**				***p***	**Girls**				***p***
	**Q1** **(20–46)**	**Q2** **(47–52)**	**Q3** **(53–57)**	**Q4** **(58–77)**		**Q1** **(20–46)**	**Q2** **(47–52)**	**Q3** **(53–57)**	**Q4** **(58–77)**	
*n* (%)	44 (20.5)	60 (27.9)	55 (25.6)	56 (26.0)		110 (28.9)	100 (26.2)	82 (21.5)	89 (23.4)	
Children age (years)	13.5 ± 2.3^a^^.^^b^	13.9 ± 2.3^a^	13.0 ± 2.4^a^^.^^b^	12.8 ± 2.4^b^	0.046	14.9 ± 2.2^a^	13.4 ± 2.6^b^^.^^d^	12.7 ± 2.1^c^	13.0 ± 2.6^d^^.^^c^	< 0.001^**^
Children BMI (kg/m^2^)	21.0 ± 4.2	20.2 ± 3.4	20.3 ± 4.0	20.2 ± 3.3	0.824	20.2 ± 3.5^a^	20.1 ± 3.9^a^^.^^b^	19.3 ± 3.5^a^^.^^b^	18.8 ± 3.5^b^	0.033^*^
Mother age (years)	42.4 ± 7.0	40.9 ± 5.1	40.3 ± 5.5	42.2 ± 6.3	0.280	42.9 ± 6.0^a^	41.1 ± 5.9^a^^.^^b^	41.2 ± 5.8^a^^.^^b^	40.5 ± 5.5^b^	0.024^*^
Mother BMI (kg/m^2^)	26.3 ± 5.6	26.1 ± 4.3	27.6 ± 5.5	27.0 ± 4.8	0.491	28.0 ± 4.8^a^^.^^c^	27.6 ± 4.3^a^^.^^b^^.^^c^	27.9 ± 4.8^c^	25.3 ± 3.9^b^	< 0.001^**^
Father age (years)	47.7 ± 7.0	46.6 ± 5.9	44.9 ± 5.7	46.2 ± 6.4	0.180	47.7 ± 5.9^a^	46.5 ± 6.8^a^^.^^b^	45.8 ± 6.9^a^^.^^b^	45.6 ± 5.8^b^	0.036^*^
Father BMI (kg/m^2^)	27.9 ± 4.1	28.2 ± 4.4	26.9 ± 3.6	28.0 ± 3.9	0.365	26.5 ± 3.8	26.6 ± 3.1	27.5 ± 4.0	27.3 ± 3.6	0.348
MET (minutes/week)	2,059.1 ± 1,704.1	1,812.1 ± 1,672.4	2,114.5 ± 1,552.1	2,268.0 ± 1,376.5	0.311	1,763.6 ± 1,522.1	1,348.2 ± 1,361.1	1,245.7 ± 1,224.2	1,868.9 ± 1,592.6	0.050

BMI, Body Mass Index; FNPA, Family Nutrition and Physical Activity Scale; MET, Metabolic Equivalent; BAZ, BMI-for-age Z score.

a, b, c The groups with the same letters within a row are not signifcantly diferent according to pairwise comparisons.

* *p* < 0.05,

** *p* < 0.001.

Child, maternal and paternal age were negatively correlated with FNPA (r = −0.217; *p* < 0.001; r = −0.103; *p* = 0.013; r = −0.124; *p* = 0.003, respectively). The education duration of the mother and father was found to be positively correlated with the FNPA score (r = 0.225; *p* < 0.001; r = 0.279; *p* < 0.001, respectively). Child and maternal BMI values were negatively correlated with FNPA score (r = −0.105; *p* = 0.011; r = −0.111; *p* = 0.008, respectively) (data not shown).

Table 4 shows daily dietary energy and NAR-MAR according to quartile categories of the FNPA. In girls, the difference between dietary energy Q4 (1,569.6 ± 454.3 kcal) and Q1 (1,737.7 ± 478.3) and MAR Q4 (80.0 ± 12.0) and Q1 (73.4 ± 15.6) was significant (*p* < 0.05). The daily dietary intake of folate, vitamin B12, vitamin C, magnesium, and iron in children was higher in Q4 than in Q1 (*p* < 0.05).

**Table 4:** Daily dietary energy.

	**Boys**				***p***	**Girls**				***p***
**FNPA quartiles**	**Q1** **(20–46)**	**Q2** **(47–52)**	**Q3** **(53–57)**	**Q4** **(58–77)**		**Q1** **(20–46)**	**Q2** **(47–52)**	**Q3** **(53–57)**	**Q4** **(58–77)**	
Dietary energy (kcal)	1,620.2 ± 399.7	1,636.3 ± 432.1	1,661.4 ± 439.1	1,610.2 ± 386.2	0.981	**1,737.7** **±478.3**^a^	1,618.5 ± 406.7^a^^,^^b^	1,645.2 ± 385.4^a^^,^^b^	**1,569.6** **±454.3**^b^	**0.029** ^*^
mean + SD										
**NAR%**										
Protein	98.1 ± 5.5	96.3 ± 9.7	97.6 ± 7.3	98.1 ± 6.9	0.880	95.8 ± 10.8	97.5 ± 7.1	98.7 ± 3.8	98.3 ± 5.4	0.486
Fiber	50.8 ± 18.0	48.8 ± 20.6	48.9 ± 17.2	51.5 ± 19.6	0.846	69.0 ± 23.3^a^^,^^b^	**61.9** **±21.1**^a^	64.2 ± 21.6^a^^,^^b^	**69.7** **±20.7**^b^	0.050
Vitamin B6	91.7 ± 13.2	90.9 ± 16.5	92.4 ± 14.7	92.0 ± 15.5	0.514	90.2 ± 16.2	91.2 ± 15.2	93.7 ± 11.6	94.3 ± 11.2	0.274
Folate	74.5 ± 21.4	72.7 ± 23.7	78.3 ± 21.9	81 ± 23	0.185	**72.8** **±23.5**^a^	76.3 ± 21.8^a^^,^^b^	80.0 ± 19.7^a^^,^^b^	**81.7** **±19.9**^b^	**0.015** ^*^
Vitamin B12	95.7 ± 13.3	88.0 ± 22.7	94.3 ± 15.3	92.4 ± 19.0	0.132	**80.2** **±31.4**^a^	90.3 ± 22.3^a^^,^^b^	92.1 ± 18.6^a^^,^^b^	**92.1** **±20.1**^b^	**0.016** ^*^
Vitamin C	84.1 ± 21.6	78.1 ± 28.5	86.5 ± 23.7	82.5 ± 25.0	0.387	**80.6** **±27.9**^a^	80.7 ± 29.5^a^^,^^b^	85.8 ± 24.8^a^^,^^b^	**90.8** **±21.3**^b^	**0.006** ^*^
Calcium	40.8 ± 19.8	40.6 ± 18.9	45.4 ± 20.4	45.7 ± 25.3	0.531	42.5 ± 21.0	44.2 ± 20.4	41.9 ± 18.1	45.9 ± 21.6	0.653
Magnesium	68.9 ± 22.2	69.9 ± 24.4	74.7 ± 24.5	76.2 ± 25.4	0.274	**70.3** **±23.5**^a^	76.6 ± 22.3^a^^,^^b^	79.9 ± 19.0^a^^,^^b^	**80.4** **±21.7**^b^	**0.012** ^*^
Phosphorus	76.3 ± 18.2	75.9 ± 17.2	78.2 ± 17.6	78.7 ± 20.3	0.742	76.0 ± 19.3	77.4 ± 18.7	73.0 ± 17.6	79.9 ± 16.6	0.080
Potassium	45.5 ± 15.9	45.0 ± 16.4	48.5 ± 16.7	48.1 ± 16.2	0.461	46.9 ± 17.8	45.6 ± 15.1	46.1 ± 16.2	51.0 ± 16.8	0.107
Iron	88.1 ± 15.8	88.6 ± 17.4	88.9 ± 16.6	90.6 ± 15.1	0.875	**73.2** **±22.1**^a^	78.3 ± 23.3^a^^,^^b^	85.3 ± 19.2^a^^,^^b^	**87.0** **±20.3**^b^	**< 0.001** ^**^
Zinc	84.7 ± 17.8	84.4 ± 18.7	86.0 ± 17.5	86.0 ± 19.5	0.766	85.0 ± 18.7	86.3 ± 17.4	85.8 ± 15.8	89.3 ± 14.8	0.403
MAR % mean+SD	74.9 ± 11.5	73.3 ± 14.5	76.6 ± 13.5	76.9 ± 15.2	0.324	**73.4** **±15.6**^a^	75.5 ± 13.6^a^^,^^b^	77.1 ± 10.7^a^^,^^b^	**80.0** **±12.0**^b^	**0.019** ^*^

NAR-MAR scores according to quartile categories of the FNPA.

NAR, nutrient adequacy ratio; MAR, mean adequacy ratio; FNPA, family nutrition and physical activity scale.

a, b, c The groups with the same letters within a row are not signifcantly diferent according to pairwise comparisons.

* *p* < 0.05,

** *p* < 0.001.

Child, maternal and paternal age were negatively correlated with MAR (r = −0.349; p < 0.001; r = −0.092; *p* = 0.026; *r* = −0.112; *p* = 0.007). Maternal education duration was positively correlated with MAR (r = 0.082; *p* = 0.046). In simple linear regression analysis FNPA score was found to be positively correlated with MAR (R2: 0.142; *p* < 0.001) (data not shown).

When the factors affecting children's BMI were evaluated with multiple linear regression analysis, the model was significant (R2: 0.358 *p* < 0.001). Children's BMI was negatively correlated with FNPA score and MAR; maternal BMI and father's BMI values were positively correlated, and MET was unrelated (*p* < 0.05) (Table 5).

**Table 5:** Multiple linear regression analysis for children's BMI prediction.

**Model**	**BMI**					

	**Beta**	**Standart error**	**t**	**p**	**Lower bound**	**Upper bound**
Constant	15.197	1.710	8.889	< 0.001	11.838	18.555
FNPA score	−0.037	0.018	−2.033	0.043^*^	−0.073	−0.001
Dietary energy	0.040	0.003	1.023	0.122	0.010	0.016
MAR	−0.035	0.011	−3.262	0.001^*^	−0.056	−0.014
MET	−0.011	0.004	−2.053	0.051	−0.020	−0.001
Mother BMI	0.093	0.031	2.985	0.003^*^	0.032	0.155
Father BMI	0.230	0.039	5.908	< 0.001^**^	0.154	0.307
			R^2^: 0.358 *p* < 0.001			

FNPA, Family Nutrition and Physical Activity Scale; MAR, Mean Adequacy Ratio; MET, Metabolic Equivalent; BMI, Body Mass Index.

* *p* < 0.05,

** *p* < 0.001.

## 4. Discussion

This study aimed to examine the effect of the family (mother and father's age, education level, BMI values, family nutrition, and physical activity habits) on the child's nutrition, BMI, and physical activity levels.

### 4.1. Family influence on children's nutritional status

In this study, the age of the child, mother, and father was negatively correlated with the MAR. Maternal education duration and FNPA score were positively correlated with MAR. It was determined that as the FNPA score increased in girls, dietary adequacy increased, and the dietary energy was lower in the group with the highest FNPA score (Q4) than in the group with the lowest (Q1). However, no difference was found between the quartiles in boys. In general, the FNPA score was found to be positively associated with MAR.

Families play a major role in the development of eating behaviors. It should be noted that a family member's eating behaviors are influenced by other members. According to the Family Systems Theory, familial influences impact children's behaviors (24). Adolescents decide on their food preferences more independently than their families (25). For this reason, the family's influence on the children's nutrition may decrease with the increase in the age of the children. At the same time, adolescence is a period of increased socialization, and the increase in food consumption preferences outside of the influence of peers may have a negative effect on dietary adequacy (26).

The literature shows that children of highly educated parents consume healthier foods (17). Parents with higher levels of education are more likely than parents with lower levels of education to be aware of healthy eating habits (17). In our study, following the literature, it was found that the children of those whose mothers had a higher education period had higher dietary adequacy scores and therefore consumed healthier diets.

Children's dietary quality strongly correlates with parental modeling of healthy eating behaviors (8). This was clear from our study's finding that children who live in healthy home nutrition and physical activity environments have healthier nutrition intake. Our findings indicate that the family may have a more effect on nutritional status in girls. The Parental behaviors subscale score was found to be significantly higher in girls. A high score can be effective in the dietary adequacy of girls. Therefore, further studies are needed on the effects of family on children's nutrition and its relationship with gender.

### 4.2. Family influence on children's BMI

In this study, child and maternal BMI values were negatively correlated with FNPA scores. According to the FNPA score in girls, the BMI value in the Q4 group was significantly lower than in the Q1. At the same time, the BMI value of the mother and father positively affects the BMI value of the child.

Pediatric health providers may utilize the FNPA to assess a child's family and home environment concerning obesity. Because it incorporates data from a variety of behaviors (such as family meals, viewing TV in bedrooms, and parental modeling of physical activity) associated with child obesity (14). In a study, the negative relations between the FNPA score with BMI, percent body fat, and BMI percentile (−0.33, −0.17, −0.29, respectively) were statistically significant (15). Also, FNPA scores in the lowest tertile had odds ratios of 1.74 (95% CI =1.05–2.91) and 2.77 (95% CI=1.22–6.27) compared with highest tertile for being overweight (15). Similar results have been shown in other studies (10, 14). The results of our study were consistent with the literature, and it was determined that a low FNPA score was associated with high BMI in children.

Children who have obese parents are at increased risk for obesity. This increased risk is partly genetic and is due to parental modeling of healthy behaviors and characteristics of the home environment (for example, access to healthy food and opportunities for physical activity) (27). In a study, parents with high BMI scores (overweight or obese) had 2.18 times the odds (95% CI 1.11–4.27) of being in the low FNPA scores (less healthy environment) (16). This study determined that mother and father BMI values were positively related to child BMI values. These results support the influence of parents on children's BMI values. Furthermore, dietary energy was not associated with the child's BMI but with dietary adequacy (MAR score). This result shows that adequate and balanced nutrition, regardless of dietary energy, may affect children's body weight.

### 4.3. Family influence on children's physical activity

In this study, it was determined that children's physical activity level was moderate in both boys and girls. It was found that 28.4% of boys and 35.7% of girls had insufficient physical activity levels. The study showed no difference in MET values according to the FNPA quartiles.

Since children learn from their parents' behaviors, it is thought that the physical activity level of the parents is related to the children's physical activity level. Children's lifestyle choices are significantly influenced by their family environment. Children's levels of physical activity were found to be significantly correlated with parental knowledge of the guidelines for physical activity, parental support for their children's physical activity, parental physical activity habits, and the availability of equipment for physical activity in the home (17). However, in this study, no difference was found in the children's physical activity levels according to their FNPA scores. The main reason may be that the study data were collected during the COVID-19 epidemic, and the children and parents could not do enough physical activity due to restrictions. The main limitation of this study is the inability to evaluate physical activity levels adequately due to the pandemic period.

There are several limitations of the study. First, the findings were based on a cross-sectional study, which makes it difficult to determine whether parenting influences a child's nutrition, BMI, and physical activity habits in a causal manner. Therefore, a longitudinal study is suggested in future research. Second, the data were based on a Turkish children sample in Erzurum city. It is suggested to replicate the study in other cities in Turkey. This restricts the conclusions of the study's generalizability. Third, parents self-reported and filled out family nutrition and physical activity pattern questionnaires, which may have led to bias. Finally, since the study was collected during the COVID-19 pandemic, it is limited in evaluating the level of the physical activity status of children.

## 5. Conclusion

The results of this study support the parenting influences on children's nutritional status and BMI. Furthermore, dietary energy was not associated with the child's BMI but with dietary adequacy. This result shows that adequate and balanced nutrition, regardless of dietary energy, may affect children's weight. Our findings indicate that the family may have more influence on nutritional status in girls. Therefore, further studies are needed on the effects of family on children's nutrition and its relationship with gender. Family plays a significant role in influencing and forming children's lifestyle-related behaviors. Children's healthy eating and physical exercise habits can be encouraged through school-based programs involving families. Understanding the moderating and mediating elements related to parenting and a child's nutritional status, physical activity, and BMI should be the focus of future research.

## Data availability statement

The original contributions presented in the study are included in the article/supplementary material, further inquiries can be directed to the corresponding author.

## Ethics statement

This research involved human participants. Ethical permission was obtained from the Erzurum Technical University Ethics Committee (Meeting Number: 10; Decision Number: 11; and 20.04.2021) and the Erzurum Provincial Directorate of National Education (31.05.2021). The study was conducted according to the rules delineated in the Helsinki Declaration, and written informed consent from the parents and child assent was obtained. Written informed consent to children in this study was provided by the participants' legal guardian/next of kin.

## Author contributions

BK-B: conceptualization, collecting the data, data analysis, writing—original draft, writing–review, and editing. SS: conceptualization, data analysis, writing—original draft, writing—review, and editing. MM-Ç: conceptualization, writing—original draft, writing—review, and editing. All authors have read and approved the final manuscript.

## References

[1] Frank LD Adhikari B White KR Dummer T Sandhu J Demlow E. Chronic disease and where you live: Built and natural environment relationships with physical activity, obesity, and diabetes. Environ Int. (2022) 158:106959. 10.1016/j.envint.2021.106959 34768046

[2] World Health Organisation. Consideration of the Evidence on Childhood Obesity for the Commission on Ending Childhood Obesity: Report of the ad hoc Working Group on Science and Evidence for Ending Childhood Obesity. Geneva, Switzerland: World Health Organization (2016).

[3] Alotaibi M Alnajjar F Cappuccio M Khalid S Alhmiedat T Mubin O. Efficacy of emerging technologies to manage childhood obesity. Diabetes Metab Syndr Obes. (2022) 15:1227. 10.2147/DMSO.S357176 35480851 PMC9037732

[4] Centers for Disease Control Prevention. Childhood Obesity Facts. Available online at: https://www.cdc.gov/obesity/data/childhood.html (accessed September 09, 2022).

[5] World Health Organization. Obesity and Overweight. (2021). Available online at: https://www.who.int/newsroom/fact-sheets/detail/obesity-and-overweight (accessed October 01, 2022).

[6] Ihmels MA Welk GJ Eisenmann JC Nusser SM. Development and preliminary validation of a Family Nutrition and Physical Activity (FNPA) screening tool. Int J Behav Nutr Phys Act. (2009) 6:1–10. 10.1186/1479-5868-6-14 19284631 PMC2669043

[7] Lee JS Jin MH Lee HJ. Global relationship between parent and child obesity: a systematic review and meta-analysis. Clin Exp Pediatr. (2022) 65:35. 10.3345/cep.2020.01620 33781054 PMC8743427

[8] Vaughn AE Martin CL Ward DS. What matters most-what parents model or what parents eat? Appetite. (2018) 126:102–7. 10.1016/j.appet.2018.03.025 29604319 PMC5971159

[9] Ozdemir S Terzi O Dundar C. The family nutrition and physical activity (FNPA) screening tool: psychometric characteristics, reliability, and validity in the Turkish population. J Public Health. (2022) 30:2525–31. 10.1007/s10389-021-01540-y

[10] Ihmels MA Welk GJ Eisenmann JC Nusser SM Myers EF. Prediction of BMI change in young children with the family nutrition and physical activity (FNPA) screening tool. Ann Behav Med. (2009) 38:60–8. 10.1007/s12160-009-9126-3 19806417

[11] Mahmood L Flores-Barrantes P Moreno LA Manios Y Gonzalez-Gil EM. The influence of parental dietary behaviors and practices on children's eating habits. Nutrients. (2021) 13:1138 10.3390/nu13041138 33808337 PMC8067332

[12] Baker S Morawska A Mitchell A. Promoting children's healthy habits through self-regulation via parenting. Clin Child Fam Psychol Rev. (2019) 22:52–62. 10.1007/s10567-019-00280-6 30725307

[13] Christofaro DG Tebar WR Mota J Fernandes RA Scarabottolo CC Saraiva BTC Delfino LD de Andrade SM. Gender analyses of Brazilian parental eating and activity with their adolescents' eating habits. J Nutr Educ Behav. (2020) 52:503–11 10.1016/j.jneb.2019.09.015 31699617

[14] Tucker JM Howard K Guseman EH Yee KE Saturley H Eisenmann JC. Association between the family nutrition and physical activity screening tool and obesity severity in youth referred to weight management. Obes Res Clin Pract. (2017) 11:268–75. 10.1016/j.orcp.2016.09.007 27717623

[15] Yee KE Pfeiffer KA Turek K Bakhoya M Carlson JJ Sharman M. Association of the family nutrition and physical activity screening tool with weight status, percent body fat, and acanthosis nigricans in children from a low socioeconomic, urban community. Ethn Dis. (2015) 25:399. 10.18865/ed.25.4.399 26675805 PMC4671432

[16] Williams JE Helsel B Griffin SF Liang J. Associations between parental BMI and the family nutrition and physical activity environment in a community sample. J Community Health. (2017) 42:1233–9. 10.1007/s10900-017-0375-y 28589267

[17] Al Yazeedi B Berry DC Crandell J Waly M. Family influence on children's nutrition and physical activity patterns in Oman. J Pediatr Nurs. (2021) 56:e42–8. 10.1016/j.pedn.2020.07.012 32811703

[18] Craig CL Marshall AL Sjöström M Bauman AE Booth ML Ainsworth BE. International physical activity questionnaire: 12-country reliability and validity. Med Sci Sports Exerc. (2003) 35:1381–95. 10.1249/01.MSS.0000078924.61453.FB 12900694

[19] Saglam M Arikan H Savci S Inal-Ince D Bosnak-Guclu M Karabulut E Tokgozoglu L. International physical activity questionnaire: reliability and validity of the Turkish version. Percept Mot Skills. (2010) 111:278–84 10.2466/06.08.PMS.111.4.278-284 21058606

[20] Lohman TG Roche AF Martorell R. Anthropometric Standardization Reference Manual. Australia: Human Kinetics Books. (1988).

[21] Onis Md Onyango AW Borghi E Siyam A Nishida C Siekmann J. Development of a WHO growth reference for school-aged children and adolescents. Bull World Health Organ. (2007) 85:660–7 10.2471/BLT.07.043497 18026621 PMC2636412

[22] World Health Organisation. Global database on Body Mass Index: BMI Classification. Geneva: World Health Organization. (2006).

[23] Mirmiran P Azadbakht L Esmaillzadeh A Azizi F. Dietary diversity score in adolescents-a good indicator of the nutritional adequacy of diets: Tehran lipid and glucose study. Asia Pac J Clin Nutr. (2004) 13:56–60. 15003915

[24] Kitzman-Ulrich H Wilson DK St George SM Lawman H Segal M Fairchild A. The integration of a family systems approach for understanding youth obesity, physical activity, and dietary programs. Clin Child Fam Psychol Rev. (2010) 13:231–53. 10.1007/s10567-010-0073-0 20689989 PMC3293190

[25] Ryan D Holmes M Ensaff H. Adolescents' dietary behaviour: the interplay between home and school food environments. Appetite. (2022) 175:106056. 10.1016/j.appet.2022.106056 35447162

[26] Salvy S-J. de la Haye K, Bowker JC, Hermans RC. Influence of peers and friends on children's and adolescents' eating and activity behaviors. Physiol Behav. (2012) 106:369–78. 10.1016/j.physbeh.2012.03.022 22480733 PMC3372499

[27] Timm A Kragelund Nielsen K Joenck L Husted Jensen N Jensen DM Norgaard O. Strategies to promote health behaviors in parents with small children—A systematic review and realist synthesis of behavioral interventions. Obes Rev. (2022) 23:e13359. 10.1111/obr.13359 34734473

